# Liquid Biopsies for Molecular Biology-Based Radiotherapy

**DOI:** 10.3390/ijms222011267

**Published:** 2021-10-19

**Authors:** Erik S. Blomain, Everett J. Moding

**Affiliations:** 1 Department of Radiation Oncology, Stanford University School of Medicine, Stanford, CA 94305, USA; blomain@stanford.edu; 2 Stanford Cancer Institute, Stanford University School of Medicine, Stanford, CA 94305, USA

**Keywords:** circulating tumor DNA, liquid biopsies, radiation biology, biomarkers, precision oncology, tumor evolution, treatment resistance

## Abstract

Molecular alterations drive cancer initiation and evolution during development and in response to therapy. Radiotherapy is one of the most commonly employed cancer treatment modalities, but radiobiologic approaches for personalizing therapy based on tumor biology and individual risks remain to be defined. In recent years, analysis of circulating nucleic acids has emerged as a non-invasive approach to leverage tumor molecular abnormalities as biomarkers of prognosis and treatment response. Here, we evaluate the roles of circulating tumor DNA and related analyses as powerful tools for precision radiotherapy. We highlight emerging work advancing liquid biopsies beyond biomarker studies into translational research investigating tumor clonal evolution and acquired resistance.

## 1. Introduction

Cancer is a heterogeneous group of diseases characterized by diverse underlying molecular changes [1]. Even within tumors arising from the same anatomical location and/or with the same histology, the underlying genetic alterations and response to therapies can vary dramatically [2,3]. Technological advances have dramatically improved the cost and availability of next-generation sequencing, leading to the wide-spread implementation of genomic and transcriptomic approaches as clinical diagnostics and tools to study human disease [4]. In particular, the field of oncology has been revolutionized by the development of personalized treatments to target oncogenic mutations and altered signaling pathways within an individual patient’s cancer [5]. As a result, precision oncology based on genomic and/or transcriptomic profiling at the time of diagnosis has become standard of care for many cancers [6]. However, recent analyses have highlighted the substantial genetic and microenvironmental heterogeneity that can exist within an individual patient’s cancer, complicating the implementation of personalized treatment approaches [7].

Although nearly half of patients with cancer receive radiotherapy, personalized treatment in radiation oncology based on patient-specific molecular profiles has lagged behind systemic therapies. There are few validated predictors of radiation response and currently no reliable approaches to monitor the response of tumors and normal tissues during radiotherapy to enable adaptive treatment approaches. As a result, radiation doses are uniformly prescribed across patients with the same stage and cancer type, and there are no combination therapies to leverage an individual patient’s molecular alterations [8].

Liquid biopsies to profile the nucleic acids released from tumors and normal tissues into the peripheral blood are transforming the management of patients with cancer [9]. In particular, tracking tumor-specific genomic alterations in the blood with circulating tumor DNA (ctDNA) is a promising approach to guide treatment decisions in patients with localized and metastatic cancer [10,11,12]. Because ctDNA can be released from all of the tumor cells within a patient, ctDNA provides unique insight into intratumoral heterogeneity. Furthermore, ctDNA can be serially analyzed from routine blood draws, enabling the dynamic study of human tumor genomics during therapy to identify mechanisms of treatment resistance [13,14,15]. Here, we review the application of liquid biopsies as biomarkers to guide precision radiotherapy. In addition, we explore the potential for ctDNA analysis and other liquid biopsy approaches to improve our understanding of molecular radiobiology in human patients.

## 2. Opportunities and Challenges for Precision Radiation Oncology

### 2.1. Advances in Precision Oncology 

An improved understanding of the genetic alterations that drive cancer development has led to the development of therapies targeting the specific molecular alterations harbored by an individual patient’s tumor [5]. Initial breakthroughs with selective estrogen receptor modulators in breast cancer [16] have expanded into therapies targeting the oncogenic proteins caused by gene fusions [17], point mutations, insertions, deletions [18], and gene overexpression [19] across diverse cancers. Increasingly, cancers are being defined by their underlying molecular alterations rather than their tissue of origin, leading to “basket” and “umbrella” clinical trials investigating targeted therapies [20]. Indeed, recent tissue-agnostic drug approvals highlight the shift to a molecular-driven approach to precision oncology [21]. In addition to genomic alterations, gene expression signatures are being increasingly harnessed for risk stratification and therapeutic decision-making [22,23]. These paradigms will continue to be refined in the coming years.

### 2.2. Challenges towards the Implementation of Molecular Biology-Based Radiotherapy

Radiotherapy has been a leader in the personalized treatment of cancer over the past century as radiation oncologists have learned to integrate clinical characteristics and anatomical features to generate highly customized radiation plans [24]. Since the implementation of 3-dimensional treatment planning, radiation oncologists, dosimetrists, and physicists have shaped radiation beams to conformally treat tumors and spare at-risk tissues. However, in contrast to contemporary medical oncology, radiation oncology has generally lacked molecular biology-driven treatment approaches. The reasons for this are multifactorial. In contrast to metastatic tumors, routine tumor genotyping is rarely performed in patients with localized disease amenable to curative-intent treatment with radiotherapy. Furthermore, local control within the radiation field (the most relevant clinical outcome for radiotherapy) has not been consistently annotated for most large genomic datasets [25], making analyses correlating radiation sensitivity and underlying molecular aberrations technically challenging. Finally, the mechanisms through which radiotherapy acts on tumors are complex and involve both cell-intrinsic and cell-extrinsic effects [26,27]. As a result, the molecular determinants of radiation response are likely multifactorial and may vary across tissues and tumor types. Ultimately, a better understanding of these factors will be critical to maximize the efficacy of radiotherapy while minimizing toxicities.

### 2.3. Genomic Alterations That Mediate Radiation Resistance

Although numerous preclinical studies have identified genes and pathways associated with cellular radiosensitivity, there are few validated genetic mediators of radiation sensitivity in human cancers. Due to their frequent mutation across tumor types, numerous studies have evaluated the association between *TP53* and *KRAS* mutations and outcomes after radiotherapy. *TP53* mutation has been associated with increased risk of locoregional recurrence in patients treated with radiotherapy for head and neck cancer [28] and pediatric sarcomas [29]. Similarly, *KRAS* mutation has been associated with worse local control after stereotactic ablative radiotherapy (SABR) for lung cancers [30,31] and liver metastases [32]. Further implicating these genetic alterations in radiation resistance, co-mutation of *KRAS* and *TP53* has been reported to correspond to a lower pathologic complete response rate after chemoradiation therapy in rectal cancer [33] and a higher rate of local recurrence after SABR for liver metastases [32]. However, other studies have not observed an association between *KRAS* and/or *TP53* mutations and radiation resistance [34,35,36], suggesting that additional factors such as other co-occurring mutations may impact the effect of these genetic alterations.

An emerging driver of radioresistance in human cancers is activation of the KEAP1/NFE2L2 pathway. *NFE2L2* encodes a transcription factor that regulates the expression of several proteins that can protect cells from radiation-induced DNA damage [37]. Activating *NFE2L2* mutations or mutations in *KEAP1,* which normally targets NFE2L2 for degradation, have been shown to predict an increased risk of local failure after radiotherapy but not surgery for lung cancer [35,38]. Remarkably, *KEAP1/NFE2L2* mutations were associated with increased local recurrence in patients treated with both chemoradiotherapy and stereotactic ablative radiotherapy (SABR), suggesting that novel combination therapies rather than dose escalation will be necessary to overcome radioresistance. 

Although many other putative genomic mediators of radiation resistance have been explored in preclinical studies, the majority of these markers have not been validated in clinical samples with relevant endpoints such as local control in the radiation field. A comprehensive discussion of these studies is outside the scope of this review, and new approaches to identify genetic alterations that mediate radiation sensitivity and resistance in patients will be crucial.

### 2.4. Gene Expression and Epigenetic Signatures to Predict Radiation Response

In addition to genomic alterations, gene expression signatures may be able to help guide precision radiotherapy [23,39]. Prospective cohorts and randomized trials have demonstrated that commercial gene expression signatures can risk-stratify patients with breast (e.g., OncotypeDx and MammaPrint) and prostate cancer (e.g., Decipher and OncotypeDx) and thereby drive clinical decision making [40,41,42,43]. Because these assays may also be associated with the risk of locoregional recurrence after surgery [44], ongoing trials are investigating the ability of commercial gene expression signatures to guide adjuvant radiotherapy decisions and treatment volumes (e.g., NCT03488693, NCT04852887). However, additional markers could be helpful to guide radiotherapy in other cancers and enable the personalization of radiation dose.

One of the first gene-expression-based models developed to predict outcomes after radiotherapy is the radiosensitivity index (RSI), which is based on the expression of 10 genes measured by microarray [45]. The RSI was developed from cell line data and was later validated in a variety of clinical cohorts [46]. The RSI has been combined with the linear quadratic model to derive the genomic-adjusted radiation dose (GARD) [47]. Numerous studies have now validated that the RSI and GARD models can predict patient survival outcomes after radiotherapy [48,49,50,51]. However, the ability of GARD to predict local recurrences in the radiation field and the optimal cutpoints across different cancers are less clear [52,53,54]. Furthermore, GARD has not been validated with RNA sequencing or formalin-fixed paraffin-embedded samples that make up the majority of clinical specimens.

Finally, epigenetic modifications such as DNA methylation could play a role in tumor radiation response [55]. Preclinical studies have suggested that DNA methylation contributes to radioresistance [56,57,58] and that radiotherapy can alter DNA methylation in cancer cells [59]. Ultimately, clinical trials will be critical to evaluate the utility of personalizing radiotherapy doses using a GARD-based or epigenetic framework.

### 2.5. Harnessing Medical Imaging to Personalize Therapy

Clinical imaging with computed tomography (CT), magnetic resonance imaging (MRI), and positron emission tomography (PET) plays a crucial role in cancer staging. The expanding field of radiomics has leveraged advancements in machine learning to extract quantitative features from radiological images and to identify associations with underlying tumor biology and patient prognosis [60]. Additionally, a number of recent studies have identified radiogenomics as a burgeoning field of study correlating radiomic information with tumor genomics [61,62]. For example, morphological, spatial, textural, and other novel features from clinical imaging may be able to determine the human papillomavirus (HPV) status of oropharyngeal squamous cell carcinomas [63,64], molecular phenotypes in ovarian cancer [65], and *TP53* mutation status in lung cancer [66]. Furthermore, radiomic signatures may be able to predict local recurrences after radiotherapy, enabling personalized radiotherapy approaches [67,68,69]. Radiomics signatures have been validated retrospectively [70,71,72], but the clinical utility of modifying radiotherapy based on these signatures has not been addressed prospectively.

### 2.6. Adaptive Radiotherapy Based on Treatment Response

An alternative to using pre-treatment predictors is personalizing radiotherapy by adapting the radiation dose and target volumes based on initial responses during treatment. To date, most efforts have focused on using mid-treatment imaging to assess response to treatment. Imaging during treatment can take the form of diagnostic scans that are acquired during treatment or scans integrated into radiation treatment delivery itself using on-board imaging. Interim PET scans currently play a critical role in early response assessment for chemotherapy in lymphomas [73,74], and recent studies have suggested that mid-treatment PET scans can assess response to radiotherapy [75,76,77]. Similarly, cone beam computed tomography (CBCT) may be helpful for assessing tumor regression during radiotherapy [78,79]. However, many tumors do not shrink substantially during treatment despite favorable responses to radiotherapy [80], and non-specific tissue changes caused by radiotherapy can complicate response measurement [81]. As a result, additional biomarkers will likely be necessary to complement mid-treatment response assessment using standard imaging.

Taken together, biologic precision radiotherapy remains in its clinical infancy. There remains a role for continued novelty and clinical translation to identify genomic predictors of radiosensitivity and biomarkers to monitor radiation response. In that context, liquid biopsies provide a unique opportunity to achieve both of these aims by providing both a highly sensitive biomarker for disease burden as well as a window into tumor biology. The remainder of this review will explore these emerging areas in greater detail. 

## 3. Liquid Biopsies to Monitor Cancer Treatment Response

### 3.1. Biology of Circulating Nucleic Acids

The use of liquid biopsies to evaluate analytes released from tumors and normal tissues into the peripheral blood may offer a solution to the challenges associated with molecular biology-based radiotherapy. Most efforts to date have focused on characterizing and tracking ctDNA, but emerging data suggest that cell-free RNA may be a complementary analyte [82]. Notably, there is also substantial interest in monitoring circulating tumor cells (CTCs) as biomarkers and predictors of cancer treatment response [83], but a detailed discussion of this approach is outside the scope of this review. The isolation of CTCs enables the evaluation of intact tumor cells and could potentially be used for in vitro assays, but CTCs are rare in patients with localized disease [9]. It is likely that the optimal non-invasive characterization of tumor biology and responses to therapy will involve combinatorial approaches in the future.

Cell-free DNA (cfDNA) was first detected from blood plasma in 1948 [84]. Since then, cfDNA has also been observed in other bodily fluids, including urine, saliva, cerebrospinal fluid, ascites, and pleural fluid [85,86,87]. Although the mechanisms of the cfDNA released into the plasma remain to be completely elucidated, the passive release of histone-bound cfDNA has been observed from normal cells and tumor cells as a result of necrosis and apoptosis with additional active and passive mechanisms likely contributing [88,89,90,91]. Histones are thought to shield DNA from nuclease degradation during these cellular death events and allow cfDNA to exist in circulation [92]. Kinetic experiments have demonstrated that the half-life of cfDNA in the plasma is less than 2 h [93,94], suggesting that ctDNA must be continuously released from tumors in order to be detected.

### 3.2. Fundamentals of ctDNA Assays

Because the majority of the cfDNA in circulation arises from normal cells [95], the detection of ctDNA relies on the identification of low-frequency tumor-specific alterations. Numerous polymerase chain reaction (PCR) and next-generation sequencing- (NGS) based approaches have been utilized for ctDNA analysis, with the inherent advantages and disadvantages described in detail elsewhere [96]. A key challenge for ctDNA analysis is separating the true tumor signal from the biologic and technical background, and technologic and bioinformatic approaches have been developed to minimize false positives [97,98,99,100]. Gene panels for ctDNA analysis can be designed to assay recurrent or clinically actionable mutations [101] or personalized to analyze an individual patient’s mutations that have been identified from an analysis of the tumor tissue [102,103]. In the metastatic setting, many commercial liquid biopsies have been approved for non-invasive tumor genotyping to identify candidates for targeted therapies [10]. Furthermore, there is a growing body of literature leveraging ctDNA analysis to screen for cancer where each patient’s mutations are unknown at the time of testing [104,105,106,107,108]. However, sensitive detection of ctDNA in localized disease or after curative-intent therapy most commonly tracks mutations identified from the sequencing of tumor tissue to minimize multiple hypothesis testing and to attain optimal limits of detection [12,99,100,109,110].

### 3.3. Predicting Cancer Relapse after Radiotherapy with ctDNA MRD

Although radiotherapy (usually in combination with chemotherapy or surgery) achieves disease remission in the majority of patients, cancer cells that are too small to be seen by conventional imaging or physical exam can persist post-treatment. The detection of this minimal residual disease (MRD) could inform treatment strategies to improve rates of cure while minimizing toxicity [111,112]. MRD-directed therapy has long been known to improve outcomes in hematologic malignancies [113,114]. Recent advancements in ctDNA analysis have made the detection of MRD feasible in solid tumors, and more than 20 studies have demonstrated that the detection of ctDNA MRD after curative-intent therapy is highly prognostic for disease recurrence [12]. After the completion of radiotherapy, ctDNA MRD has been shown to predict outcomes across a variety of cancers, including lung cancer [115,116], breast cancer [117], rectal cancer [118,119], and esophageal cancer [120].

It is a widely held hypothesis that ctDNA MRD analysis may allow the personalization of adjuvant therapy to improve the probability of treatment cure. A number of published cases have demonstrated that adjuvant chemotherapy can reduce ctDNA levels following definitive therapy [103,121,122,123]. Despite this promising preliminary data, the substantial false negative rates of ctDNA MRD landmark analysis with most current approaches [12] may indicate that ctDNA MRD might mainly detect patients with the highest residual disease burden who are poorer candidates for treatment escalation. To address this question, we recently retrospectively compared patients treated with and without consolidation immunotherapy after chemoradiotherapy for non-small cell lung cancer. We only observed a significant benefit in freedom from progression for consolidation immunotherapy in the subset of patients with ctDNA MRD detected at the end of definitive therapy [115]. In line with these findings, a recent exploratory analysis of the IMvigor010 study evaluating adjuvant atezolizumab in muscle invasive urothelial cancer only demonstrated an overall survival benefit in the subset of ctDNA positive patients [124]. Several ongoing interventional clinical trials will help to address the clinical utility of ctDNA MRD-directed adjuvant therapy [12]. However, recent Medicare approvals for ctDNA MRD testing have opened the door to widespread clinical implementation.

### 3.4. Association of Mid-Radiotherapy ctDNA Levels with Outcomes

**Figure 1:**
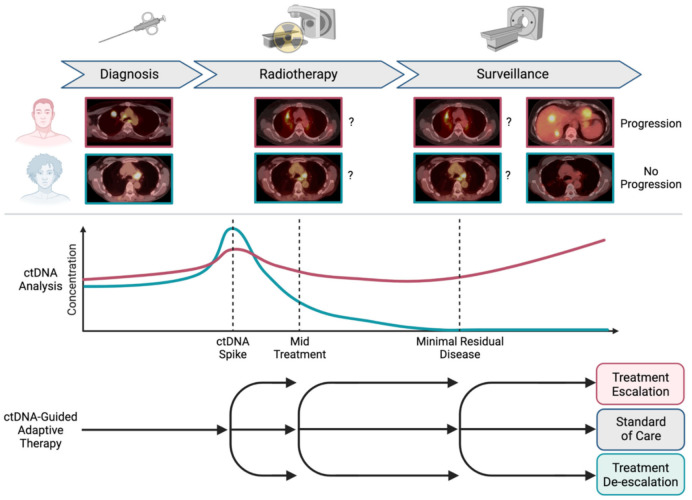
Tracking radiotherapy response using circulating tumor DNA (ctDNA) analysis. Two patients with cancer undergo definitive radiotherapy, with one patient ultimately progressing (pink) and the other patient being cured (teal). Standard radiographic imaging during radiotherapy and follow up is inconclusive due to radiographic similarities between viable tumor and other benign processes such as inflammation. In contrast, ctDNA changes during radiotherapy may identify early and mid-treatment ctDNA changes associated with response to treatment. After completing radiotherapy, ctDNA minimal residual disease can identify patients who are at risk of relapse. Escalating or de-escalating treatment based on ctDNA levels may improve the probability of cure while minimizing the risk of toxicity.

Levels of ctDNA correlate with tumor burden on imaging and prognosis pre-treatment [97,103,116,125,126], suggesting that absolute ctDNA levels or ctDNA kinetics during treatment could be used to monitor radiotherapy response and to enable adaptive treatment approaches (Figure 1). Although there is ample precedent for ctDNA kinetics predicting the response to systemic therapy [127,128,129,130,131,132], the effects of radiotherapy on ctDNA levels are just starting to be explored. Due to the association of cfDNA release with cell death, it will be critical to distinguish the rising ctDNA levels associated with radiation-induced tumor killing from decreased tumor burden during the course of therapy. Furthermore, levels of normal cfDNA increase with tissue injury and inflammation [133,134], so it may be necessary to account for the effects of radiation on normal tissues. 

After the irradiation of mouse xenograft models with 20 Gy, ctDNA levels peaked 96–144 h after radiotherapy [90], suggesting that early ctDNA kinetics could be useful for monitoring radiation-induced tumor cell killing. Early clinical data have also suggested that ctDNA levels transiently rise after starting radiotherapy. In Epstein Barr virus (EBV)-associated nasopharyngeal cancer where EBV DNA levels have been used as a biomarker analogous to ctDNA, EBV levels were observed to increase within the first week of radiotherapy before ultimately falling off, likely reflecting decreased tumor burden and treatment response [135]. In one small study of five patients with non-small cell lung cancer treated with radiotherapy alone, two patients showed a transient increase of ctDNA at 72 h, with a trend towards decreased ctDNA in all patients at 7 days [136]. Expanding these findings to larger cohorts with more frequent blood collections will be necessary to characterize early ctDNA kinetics during radiotherapy and to determine if a larger “spike” is associated with improved local control and survival outcomes.

Few studies to date have examined whether ctDNA levels at later time points (greater than one week) during conventionally fractionated radiotherapy are associated with patient outcomes. A study of 47 rectal cancer patients treated with chemoradiotherapy found no significant association between ctDNA levels 3–4 weeks from the start of treatment and patient outcomes [119]. However, 79% of patients had undetectable ctDNA midway through chemoradiotherapy despite only 13% of patients experiencing a pathologic complete response, suggesting that more sensitive analysis approaches may be necessary to identify patients with persistent local disease. We recently reported that ctDNA concentration a median of 3 weeks into chemoradiotherapy for lung cancer was significantly associated with risk of disease progression and overall survival [137]. Furthermore, integrating ctDNA analysis with complementary outcome predictors may improve risk stratification [138]. These observations will ultimately need to be evaluated prospectively, and interventional clinical trials will be necessary to confirm the utility of adapting treatment based on mid-radiotherapy ctDNA levels.

If mid-radiotherapy ctDNA levels can be proven to be a reliable predictor of patient outcomes, it will be important to determine the best approach to adapt radiotherapy. One conceptual approach would be to modify radiation dose based on treatment response. Historical dose escalation studies have illustrated that radiation doses lower than the current standard of care can achieve local control in a subset of patients [139,140]. In addition, radiation dose de-escalation is an area of active investigation in patients with HPV-positive oropharyngeal cancer [141] and has been shown to be feasible in patients with indolent and aggressive non-Hodgkin lymphoma [142]. As mentioned above, dose escalation with SABR was not sufficient to overcome the radiation resistance associated with *KEAP1/NFE2L2* mutations in lung cancers [35], and recent randomized radiation dose-escalation studies have failed to demonstrate a local control benefit in unselected cancers [143,144,145]. However, it is possible that a subset of patients could benefit from radiotherapy dose intensification. Alternatively, treatment could be adapted by changing concurrent systemic therapy to simultaneously address tumors within the radiation field and micrometastatic disease contributing to persistently elevated ctDNA levels during radiotherapy. Although a long way from clinical implementation, these approaches represent a powerful future direction for personalized radiotherapy.

### 3.5. cfDNA and Circulating RNA to Detect Gene Expression and Normal Tissue Injury 

To date, most analysis of circulating nucleic acids has focused on tracking genetic alterations, but novel approaches are under development to detect DNA features such as methylation and fragmentation patterns (i.e., fragmentomics) [146]. Furthermore, previous reports have suggested that cancer cells [147,148] and normal tissues [82,149] release RNA into circulation. Because these non-genetic signatures are unique to their cells of origin, they could provide a novel marker of tissue-specific pathologies. There is growing interest in using these new approaches as cancer biomarkers that may be complementary to tracking tumor mutations [82,106,150,151,152,153,154,155]. Furthermore, cell-free DNA epigenetic markers have been shown to be associated with normal tissue injury [92,156,157,158], raising the possibility that cell-free DNA methylation, fragmentomics, and/or cell-free RNA could identify radiation-induced normal tissue toxicity. In that context, a recent study demonstrated that levels of circulating *RRM1* RNA may be associated with risk of oral mucositis during radiotherapy for head and neck cancers [159]. These emerging data provide compelling rationale for non-invasive longitudinal monitoring of both tumor and normal tissue responses to radiotherapy to enable precision radiation oncology.

## 4. Studying Molecular Radiobiology with Liquid Biopsies

### 4.1. Unique Insights into Tumor Heterogeneity and Clonal Evolution

In addition to being a powerful biomarker to enable personalized cancer therapy, liquid biopsies provide a unique window into tumor biology that can be sampled at multiple time points over the course of therapy. Recent studies have highlighted the substantial genetic heterogeneity that can exist with human tumors [3]. The accumulation of mutations, genetic drift, and selective pressures within the tumor microenvironment can lead to tumor evolution that can impact how cancers respond to treatment [7]. Multi-region sequencing has suggested that early tumor development may occur under weak evolutionary pressures [160,161] but that selection appears to increase at later stages of tumor development and varies across tumor types [162,163,164]. The impact of intratumoral heterogeneity on radiotherapy outcomes has not been explored in detail, but modeling experiments suggest that subclones with heterogenous radiosensitivity could have a large impact on the ability of radiotherapy to control cancers [165]. Although there is strong evidence that systemic therapy selects resistant subclones that lead to disease progression [166], the effect of radiotherapy on tumor evolution is less clear. The sequencing of pre- and post-treatment samples from patients undergoing chemoradiotherapy for rectal cancer suggested that radiotherapy can substantially impact the clonal architecture and can potentially select resistant subclones [167]. Corroborating these findings, retrospective clinical studies have reported worse local control after reirradiation compared to initial treatment with radiotherapy [168,169], but these studies are confounded by lower doses of radiation in the reirradiation setting due to concern for toxicity. 

**Figure 2:**
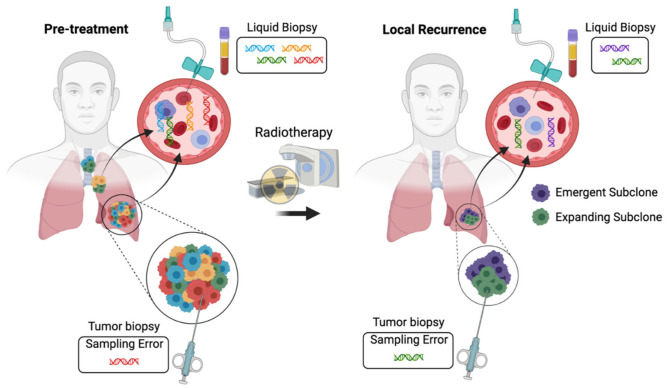
Interrogating tumor heterogeneity, clonal evolution, and resistance to radiotherapy using liquid biopsies. Tumors consist of multiple subclones with distinct genetic alterations. Tumor biopsies sample a single region of one tumor deposit. As a result, resistant subclones may be missed due to sampling error. In contrast, liquid biopsies may characterize the full extent of intratumoral heterogeneity by sampling circulating tumor DNA released from all of the tumor deposits within a patient. Liquid biopsies can also be repeated at multiple time points, enabling the longitudinal tracking of clonal evolution during radiotherapy and the identification of emergent and expanding tumor subclones that mediate radiation resistance.

Two major limitations to studying clonal evolution during radiotherapy are (1) sampling biases related to analyzing one or a few regions within a patient’s tumor and (2) a lack of longitudinal specimens to study clonal dynamics. ctDNA has the potential to be released from all of the tumor cells in a patient and can be sampled longitudinally from routine blood draws, making it a powerful tool to characterize clonal evolution (Figure 2). Multiple studies have applied ctDNA analysis to monitor clonal evolution during the treatment of patients with systemic therapy. For example, analysis of paired pre-treatment and end-of-treatment ctDNA samples demonstrated clear evidence of resistant subclone selection during the treatment of metastatic estrogen receptor-positive breast cancer with CDK4/6 inhibition and endocrine therapy [170]. In addition, ctDNA analysis demonstrated that subclonal tumor populations contribute to relapse after surgery and adjuvant chemotherapy for early stage lung cancer [103]. As a result, longitudinal ctDNA analysis and subclone tracking could help to clarify the modes of tumor evolution during radiotherapy.

### 4.2. Identifying Mediators of Radiotherapy Resistance and Sensitivity

Due to the challenges described above associated with identifying genetic alterations that mediate radiation response, innovative approaches will be necessary to identify novel mediators of radiotherapy resistance that can inform precision therapy. By enabling the longitudinal analysis of tumor genomics, liquid biopsies can help to identify expanding and emergent mutations that may contribute to treatment resistance. For example, a prospective study of patients undergoing targeted therapy for gastrointestinal cancers found that ctDNA analysis identified resistance alterations not identified from matched tumor sequencing in 78% of cases [171]. Furthermore, multiple resistance alterations were identified in 40% of patients by ctDNA analysis compared to only 9% of patients by matched tumor sequencing. Several additional studies have also leveraged ctDNA analysis to identify mechanisms of resistance to systemic therapy [13,14,132,172]. Integrating these approaches with cell-free DNA methylation profiling and/or cell-free RNA analysis could help to dissect the molecular mechanisms of resistance to radiotherapy in human cancers.

In most cancers, the first assessment of treatment response after radiotherapy does not occur until months after the completion of treatment, making it challenging to identify rapid responders. The radiation-induced inflammatory and fibrotic tissue changes described above also complicate early response assessment to radiotherapy with imaging. ctDNA kinetics during treatment represent novel markers of treatment response that may enable the identification of rapid responders [173]. Characterization of these ctDNA rapid responders could help to identify the molecular signatures that underly radiosensitivity to enable the pre-treatment identification of patients who will respond favorably to treatment.

## 5. Conclusions

Liquid biopsies have arrived in clinical practice as a non-invasive approach to monitor tumor burden and to enable personalized treatment approaches. Clinical applications of ctDNA testing in radiation oncology such as mid-treatment response assessment have just started to be explored and have the potential to enable new approaches for precision radiotherapy. Beyond monitoring treatment response, liquid biopsies are a powerful tool to interrogate tumor and radiation biology. Analyzing circulating nucleic acids has the potential to provide critical insights into tumor heterogeneity, clonal evolution, and the genomic signatures that determine how individual patients respond to radiotherapy. Going forward, it will be important to balance the hypothesis-generating studies discussed here with prospective clinical trials that can shift the paradigm of patient management towards precision radiotherapy based on tumor molecular biology.

## Data Availability

Not applicable.
